# Osteosarcoma of the Proximal Fibula: A Case Report and Review of the Literature

**DOI:** 10.7759/cureus.38195

**Published:** 2023-04-27

**Authors:** Oğuzhan Muslu, Tolgahan Cengiz, Şafak Aydın Şimşek, Ahmet Ersoy, Bedirhan Albayrak, Hüseyin Sina Coşkun, Nevzat Dabak

**Affiliations:** 1 Orthopaedics and Traumatology, Hatay Training and Research Hospital, Hatay, TUR; 2 Orthopaedics and Traumatology, Ondokuz Mayıs University Faculty of Medicine, Samsun, TUR

**Keywords:** primary bone neoplasm, lateral collateral ligament, peroneal nerve, osteosarcoma, proximal fibula

## Abstract

Osteosarcoma is the most common primary malignant bone tumor, especially in younger patients. Diagnosis is based on the combined evaluation of radiological, clinical, and pathological examinations. It is usually located in the distal femur, proximal tibia, and proximal humerus. The fibula is a rare localization for osteosarcoma. Surgery in this region is challenging due to the complex anatomic structures around the knee. Especially the peroneal nerve, lateral collateral ligament (LCL), and popliteal vessel branches are of critical importance. However, additional structures such as the arcuate ligament, biceps femoris, and iliotibial band play an essential role in the stabilization of the knee. Thus, these structures must be protected as much as possible. This case report aims to present the diagnosis and treatment process of conventional osteosarcoma in the proximal fibula, which was located close to the peroneal nerve and required LCL reconstruction after the resection.

## Introduction

Osteosarcoma is a malignant bone tumor characterized by the osteoid structure of atypical malignant osteoblasts and is seen most often in the second decade of life. It is the most common of the primary malignant bone tumors, and when the etiology is examined, the majority are primary. However, it may also be seen secondary to Paget’s disease, radiation exposure, trauma, chemotherapy, or foreign body [1]. Patients generally present with complaints of pain and swelling. Definitive diagnosis is reached by radiological and pathological evaluation.

Osteosarcoma is generally seen in the distal femur, proximal tibia, and proximal humerus. The proximal fibula is a rare localization for primary osteosarcoma, with the rate previously reported in the literature being about 2% [2]. The proximal fibula is a surgically challenging region due to the close relationship of neurovascular structures. Depending on this relationship between the vascular and nerve structures and the tumor, these structures are preserved or excised. Surgical margins in thin tubular bones such as the fibula may extend more proximal or distally, contrary to what appears on MRI. Thus, wide resection with wider margins must be considered to achieve negative surgical margins. Pathological examination is recommended for determining intraoperative surgical margins. This case report presents the diagnostic approach and treatment algorithm of an 18-year-old male patient with proximal fibula localization of conventional osteosarcoma.

## Case presentation

An 18-year-old male patient presented at our center with complaints of pain and palpable swelling in the right knee. The patient also described numbness along the course of the peroneal nerve from the knee to the ankle. There was nothing remarkable in history. Physical examination revealed a full active-passive range of movement in the right knee joint, which was painful at the upper limits. The vascular examination was regular, but there was determined to be hypoesthesia in the peroneal nerve dermatome area in the neurological examination.

**Figure 1:**
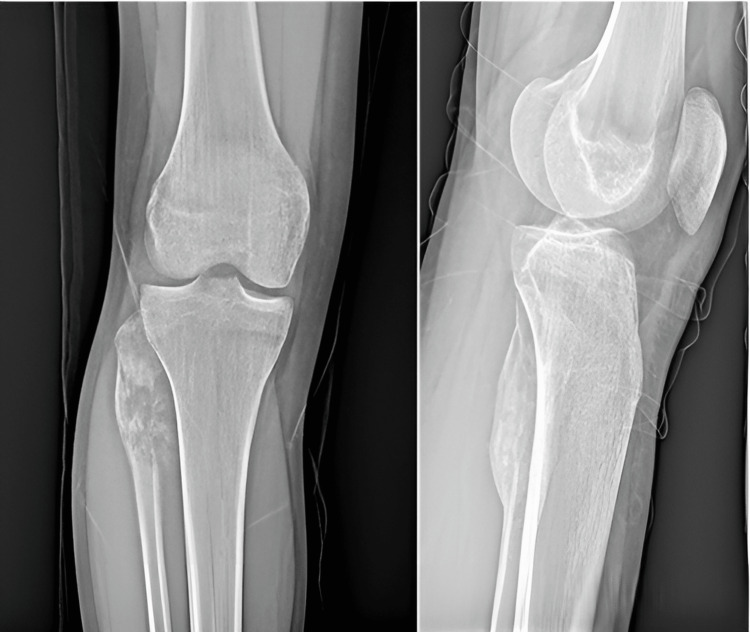
Direct radiograph of the patient who presented with complaints of pain and swelling in the right knee A lesion can be seen containing opacities and showing a periosteal reaction, which was destroying the cortex in the proximal fibula.

**Figure 2:**
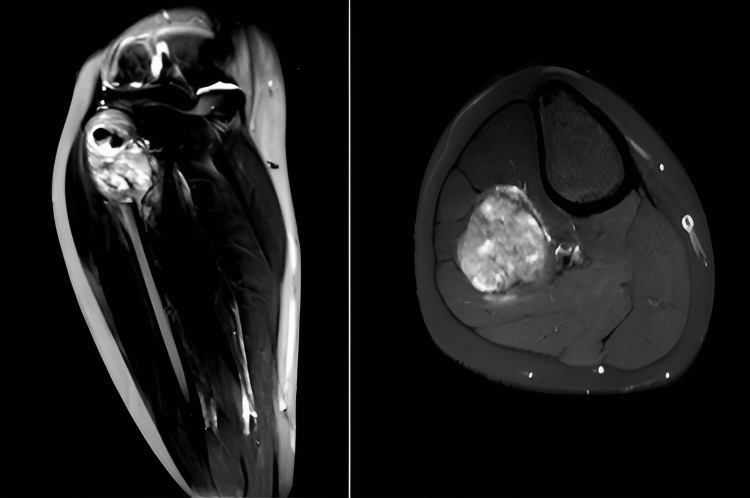
MRI showing a mass consistent with osteosarcoma localized in the proximal fibula and showing soft tissue expansion

On the plain radiograph (Figure 1), a lesion was determined which was destroying the cortex of the right fibula head, showing periosteal reaction and containing opacities. Computed tomography and MRI were performed to examine the relevant region further (Figure 2).

**Figure 3:**
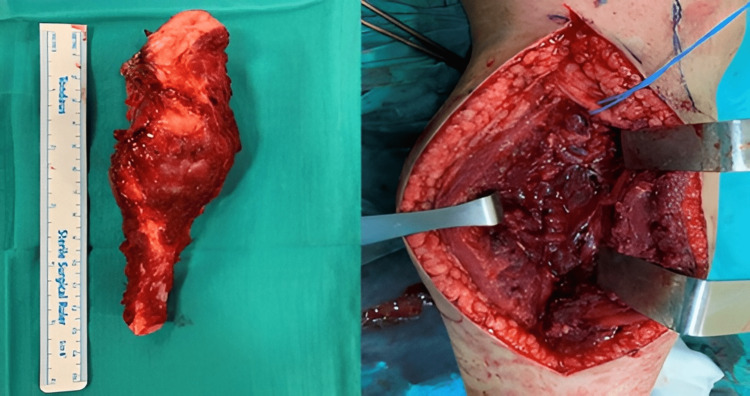
Wide resection was performed to the proximal fibula, preserving the peroneal nerve.

**Figure 4:**
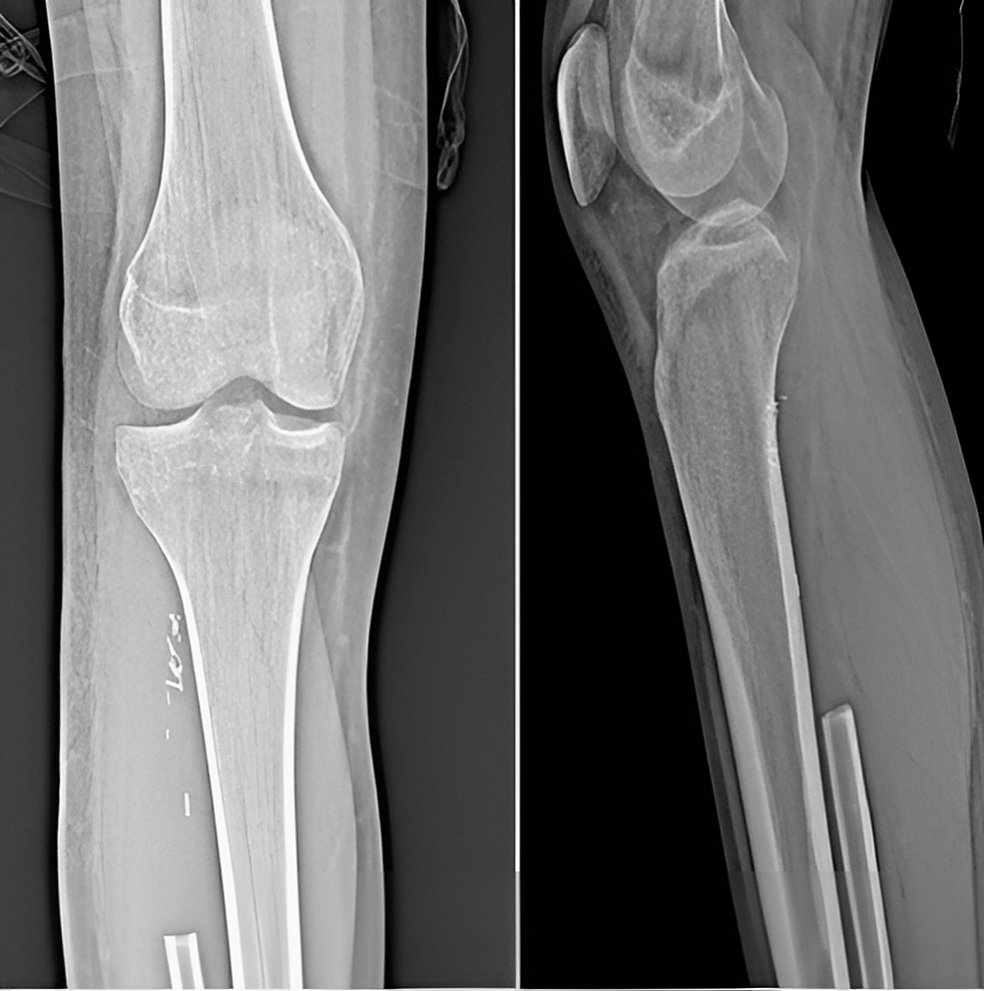
Radiograph of the patient at the one-month postoperative follow-up

After these examinations, the multidisciplinary bone and soft tissue tumor council evaluated the patient. The lesion was first thought to be a malignant bone tumor, probably osteosarcoma. Therefore, the biopsy was planned for the patient. To determine possible metastasis, positron emission tomography (PET)-CT scans were used. The biopsy results were reported as conventional osteosarcoma, and the pediatric oncologist recommended neo-adjuvant therapy. A methotrexate, adriamycin, and cisplatin (MAP) protocol was started. Following the completion of neo-adjuvant chemotherapy, PET scans were used, and no metastasis was determined. In the preoperative MRI of the area taken, a heterogeneous densely enhanced lesion with dimensions of approximately 6x3x3.5 cm, extending from the right fibula proximal metaphysis to the diaphysis, destroying the cortex and extending to the soft tissue, was observed. The closest neighborhood of the lesion to the tibial artery and peroneal nerve was measured at 2 cm. This osteosarcoma originating from the fibular head was located in the superficial posterior and lateral compartments of the cruris and extended to the shaft of the fibula. Thus wide surgical resection was planned. With a lateral approach over the mass, an incision was made to include the previous biopsy tract and wide resection was performed around the proximal fibula, preserving the peroneal nerve (Figure 3). The procedure was terminated because the surgical margins were negative in the intraoperative frozen examination. Posterolateral ligament reconstruction couldn’t be performed, but the lateral collateral ligament (LCL) was transferred to around the Gerdy tubercle in the proximal tibia following fibula resection. At the one-month postoperative follow-up examination of the patient, the complaints from the first presentation were determined to have dramatically improved (Figure 4). The paediatric oncology department initiated the adjuvant treatment for the patient following the operation. Following surgical treatment, PET was rescheduled to detect if there was any local recurrence. In the postoperative follow-up of the patient, no recurrence was found, and there was no pathological contrast enhancement on the control MRI.

## Discussion

Conventional osteosarcoma is seen most often in the second decade of life. Localization is seen most frequently in the bones around the knee joint, usually in the distal femur and proximal tibia, and it may also be seen in the proximal humerus. It is seen more often in males than females at a ratio of 3:2 [3]. Until proven otherwise, the possibility of osteosarcoma should be kept in mind in adolescents with pain adjacent to major joints, especially around the knee. Patients generally present with complaints of pain and swelling. Diagnosis is established by evaluating radiological and pathological examinations. The five-year survival rate in cases of conventional osteosarcoma with extremity localization without metastasis has been reported to be approximately 65% [4].

The surgical treatment of osteosarcoma cases with proximal fibula localization is challenging because of the peroneal nerve course along the surgical site. In cases where the peroneal nerve is preserved, the surgical margins may remain insufficient to obtain a tumor-free region. In a series of 13 cases published by Takahashi et al. [5], it was reported that the peroneal nerve was preserved in the first surgery of four cases, but local recurrence developed. The main goal of malignant bone tumor surgery is to obtain negative surgical margins. Preserving the peroneal nerve is always a concern for the surgeon, but this idea forms a barrier to obtaining adequate surgical margins in such cases. Following the wide resection, the negative surgical margins were confirmed in the current case with a frozen examination. Finally, it depends on the surgeon’s experience to preserve the nerve or not. The nerve must be sacrificed if the surgeon is unsure about the negative surgical margins. The main goal of malignant bone tumor surgery is to obtain negative surgical margins.

Furthermore, in this case, the tumor can be classified as type IIB according to Enneking classification. Firstly, the peroneal nerve was dissected by protecting the nerve. If the peroneal nerve had passed through the lesion, it would have to be removed along with the lesion. Our resection is similar to Malawer type I, we also preserved neurovascular structures including the peroneal nerve and anterior tibial artery. Amputation should be considered if limb salvage surgery can not achieve adequate surgical margins [6].

In a series of three cases with osteosarcoma involving the proximal fibula, Lushiku et al. performed wide resection to the fibula. The survival and functional outcomes of all three cases were reported to be promising [7]. Saine et al. reported that survival was directly related to wide resection applied to the proximal fibula in eight cases [2].

The critical anatomic structures that must be considered in proximal fibula resection are the LCL, arcuate ligament, biceps femoris, and iliotibial band. These structures play an essential role in stabilizing the knee [8]. Einoder (&) Choong reported that the knee remained stable following proximal fibula resection performed without LCL reconstruction [9]. In the current case, the LCL was transferred to around the Gerdy tubercle in the proximal tibia following fibula resection. The same concern on obtaining negative surgical margins also rules when protecting ligamentous structures. The main goal is always to achieve adequate surgical margins. In the follow-ups, no ligamentous instability was observed in the patient.

The metastatic spread of the disease is primarily to the lungs (80% to 90%) via the venous route [3]. With the medical oncology department's evaluation, chemotherapy is administered to the patient with a high percentage of necrosis to prevent local recurrence after surgical treatment [10,11]. Therefore, follow-up and treatment of a patient after surgery must be managed with a multidisciplinary approach. In the current case, the paediatric oncology department initiated mifamurtide as an adjuvant therapy for the patient in the postoperative period.

In bones with thin lumens, tumor margins may occur more proximal than those detected on MRI. Fibula is a model bone for this situation. In our case, we performed an additional 2 cm fibular resection from the tumor area detected on the MRI. Thus intraoperative frozen section should be planned during the surgical planning to confirm the negative surgical margins. For this reason, careful wide resection should be performed as in all malignant bone tumors with a thin bone lumen.

## Conclusions

Proximal fibula localization is an uncommon localization for osteosarcoma. Neurovascular structures and ligamentous structures cause challenges in surgical treatment. When preserving the peroneal nerve, insufficient resection may be made, and it must be kept in mind that local recurrence can result from this. The primary aim in these types of malignant tumors must be wide and sufficient resection. 
